# Overexpression of Ubiquinol-Cytochrome c Reductase Core Protein 1 May Protect H9c2 Cardiac Cells by Binding with Zinc

**DOI:** 10.1155/2017/1314297

**Published:** 2017-06-06

**Authors:** Tingting Yi, Xiaoxiao Wu, Zonghong Long, Guangyou Duan, Zhuoxi Wu, Hong Li, Huifang Chen, Xiaoying Zhou

**Affiliations:** ^1^Department of Anesthesiology, Xinqiao Hospital, Third Military Medical University, Chongqing 400037, China; ^2^Department of Anesthesiology, Yongchuan Hospital, Chongqing Medical University, Chongqing 402160, China

## Abstract

In several recent studies, proteomics analyses suggest that increase of ubiquinol-cytochrome c reductase core protein 1 (UQCRC1) is cardio-protective. However, direct evidence for this effect has not yet been obtained. Thus, the current study aimed to determine this effect and the mechanism underlying this effect. The results showed that overexpression of UQCRC1 protected H9c2 cardiac cells against in vitro simulated ischemia-reperfusion by maintaining mitochondrial membrane potential and suppressing the expression of caspase-3. These protective effects were significantly enhanced by exogenous Zn^2+^ but completely abolished by Zn^2+^-selective chelator TPEN. Furthermore, the upregulation of UQCRC1 reduced the concentration of free Zn^2+^ in mitochondria, whereas the downregulation of UQCRC1 increased the concentration of free Zn^2+^ in mitochondria. In conclusion, the overexpression of UQCRC1 can protect H9c2 cardiac cells against simulated ischemia/reperfusion, and this cardio-protective effect is likely mediated by zinc binding.

## 1. Introduction

Although considerable efforts have aimed to better protect patients with coronary heart disease against myocardial ischemia/reperfusion injury, coronary heart disease remains the leading cause of morbidity and mortality worldwide, with more than 7 million deaths per year [1]. Recently, proteomics analyses in several studies have suggested that changes in some mitochondrial proteins are involved in cardio-protection [2–5]. Of these mitochondrial proteins, ubiquinol-cytochrome c reductase core subunit 1 (UQCRC1) is frequently indicated. UQCRC1 is a subunit of complex III, which is a component of the mitochondrial electron transport chain [6–8].

Previous studies have found that UQCRC1 expression is decreased in isolated rat hearts after ischemia-reperfusion (I/R). [5] However, its expression is increased when cardio-protection is induced by glycogen synthase kinase (GSK) inhibitor VIII [2]. Furthermore, UQCRC1 overexpression enhanced complex III activity in mice [9], and its loss was associated with significant mitochondrial dysfunction in cells of epithelial origin [10]. This finding suggested that UQCRC1 contributes to normal mitochondrial function, which is essential for the cardio-protective effects induced by ischemic preconditioning (IPC) and postconditioning [11]. Therefore, the upregulation of UQCRC1 has been speculated to contribute to cardio-protection. However, direct evidence for the specific role of UQCRC1 in cardio-protection is currently unavailable.

Additionally, UQCRC1 may contain a Zn^2+^ binding site [12], which has been demonstrated to contribute to the cardio-protective effect induced by IPC, postconditioning, and pharmacological preconditioning [13–18]. Early studies found that Zn^2+^ can reversibly inhibit the electron transfer of mitochondrial complex III by binding to residues in the vicinity of the iron-sulfur protein (ISP) [19, 20]. Because UQCRC1 is located near NH_2_-terminus of the ISP [12], UQCRC1 may interact with mitochondrial Zn^2+^, and this interaction may contribute to the observed cardio-protective effect. Taken together, we hypothesize that the overexpression of UQCRC1 provides a cardio-protective effect by binding mitochondrial Zn^2+^. Therefore, the current study aimed to explore the effect of UQCRC1 overexpression on cardio-protection and the mechanism underlying this effect, with a focus on the interaction between UQCRC1 and Zn^2+^.

## 2. Materials and Methods

### 2.1. Cell Culture

The H9c2 cell line (rat embryonic ventricular myocytes) was purchased from the purchased from the Cell Bank of the Chinese Academy of Sciences (Shanghai, China). The cells were cultured in Dulbecco's modified Eagle's medium supplemented with 10% fetal bovine serum (FBS) and 100 units of penicillin-streptomycin at 37°C in a humidified 5% CO_2_ + 95% air atmosphere.

### 2.2. Adenovirus Infection

Ad-UQCRC1 was prepared and purified using protocols similar to those described in previously reported studies [21, 22]. Briefly, an E1-deleted replication-deficient adenoviral vector carrying the UQCRC1 gene linked to a green fluorescent protein (GFP) was constructed using a proprietary kit (Toyobo life science, Japan). The UQCRC1 cDNA and a human cytomegalovirus promoter were inverted into a shuttle plasmid and cotransfected into 293T cells. After recombination, plaque isolates were screened, and the selected clone was transfected into 293T cells and purified by three rounds of discontinuous CsCl step-gradient centrifugation. The titer for the Ad-UQCRC1 preparation was 4 × 10^9^ plaque-forming units/ml.

H9c2 cardiac cells were infected with the adenovirus carrying the UQCRC1 gene linked to a GFP at a multiplicity of infection (MOI) of 50 plaque-forming units per cell in PBS for 2 hours in 95% air and 5% CO_2_ at 37°C, followed by the addition of fresh medium containing FBS. The incubation was for an additional 22 hours. The medium was replaced 24 hours after the infection with DMEM containing 10% FBS and incubated for another 24 hours prior to treatment. The efficiency of adenoviral gene transfer (Ad-UQCRC1) was evaluated according to the number of cells showing a green fluorescence signal. H9c2 cardiac cells infected with Ad-GFP were used as a control, and the results showed that nearly 100% of cardiac cells appeared to be successfully infected 24 hours after the infection.

### 2.3. Western Blot Analysis

The expression level of UQCRC1 was examined by Western blotting. To this end, cells were harvested 24 hours after adenovirus infection, and equal amounts of protein (50–150 *μ*g) were analyzed by SDS-PAGE for each group. After transferring the protein to membranes, an immunoblotting analysis was performed using the primary antibodies against UQCRC1 and GAPDH. The binding of each primary antibody was detected with a secondary antibody at a 1 : 2000 dilution and visualized using the enhanced chemiluminescence (ECL) method. The intensities of antibody complexes were assessed with the Image Pro-Plus 5.1 software, and the protein expression indicated by the band intensity was expressed as the percentage of the control group.

### 2.4. Oxygen and Glucose Deprived/Reoxygenation (OGD/R) Injury Model

The OGD/R model in the current study was established following previously described protocols [23–25]. Briefly, H9c2 cells were cultured to 50–60% confluence and infected with Ad-UQCRC1 or Ad-GFP as previously described. The medium was then replaced with DMEM without FBS 12 hours before the OGD/R injury model was initiated. After 12 hours of incubation, the cultured cells were washed twice with standard Tyrode solution containing (mM) 140 NaCl, 6 KCl, 1 MgCl_2_, 1 CaCl_2_, 5 HEPES, and 5.8 glucose (pH 7.4) and incubated in the standard Tyrode solution for 2 hours before the following experiments. To induce OGD/R, the cells were cultured in glucose-free Tyrode solution containing 10 mM 2-deoxy-D-glucose and 10 mM sodium dithionite and transferred to a humidified hypoxia chamber (International Stem Cell Corporation, California, USA). Subsequently, the cells were flushed with 95% N_2_ + 5% CO_2_ to generate a hypoxic environment, and the sealed chamber was placed into a 37°C incubator for 1 hour. After the OGD incubation, the cells were exposed to fresh medium and incubated in 95% air + 5% CO_2_ for 2 hours.

### 2.5. Cell Viability Assay

The cell viability was assessed with a CCK-8 (cell counter kit 8) assay, a sensitive nonradioactive colorimetric assay for measuring cell growth (DojindoLab., Japan), according to the manufacturer's instructions and previous studies [26, 27]. Briefly, H9c2 cells were plated in 96-well plates. After the OGD/R, CCK-8 solution (10 *μ*L) diluted 1 : 10 with DMEM (100 *μ*L) was added to each well. The plates were then incubated at 37°C in a humidified 5% CO_2_ + 95% air atmosphere for 2 hours before measuring the absorbance at 450 nm using an enzyme-linked immunosorbent assay microplate reader (Thermo Scientific). The results are reported as the percentage of the control group.

### 2.6. Cell Apoptosis Assay

The percentage of apoptotic or necrotic cells was assessed using an Apoptosis and Necrosis Assay Kit (Beyotime, China) according to the manufacturer's instructions and a previous study [28]. Briefly, H9c2 cells were cultured in 24-well plates at a concentration of 9,000 cells/well. After 48 hours of adenovirus infection, the cells were treated with 200 *μ*M H_2_O_2_ for 20 min, while the control cells remained untreated. The cells were then stained with both Hoechst 33342 and propidium iodide (PI) for 20 min at 4°C in the dark and examined using fluorescence microscopy. The nuclear morphology was analyzed based on the fluorescent DNA-binding dye Hoechst 33342, and the necrotic cells were identified by propidium iodide, which is commonly used to mark necrotic cells because it is membrane-impermeable and generally excluded from viable cells. The numbers of apoptotic or necrotic cells were analyzed using the Image Pro-Plus software 5.1 and reported as the percentage of total cells at the end of the experiment. At least 1,000 cells from each group were counted and distinguished as viable, apoptotic or necrotic cells.

### 2.7. Zn^2+^ Regulation

H9C2 cells were divided into four groups based on treatment: the Ad-GFP and Ad-UQCRC1 groups were infected with Ad-GFP or Ad-UQCRC1 as described above, whereas the Ad-UQCRC1+Zn^2+^ and Ad-UQCRC1+TPEN groups consisted of Ad-UQCRC1 cells treated with 1 *μ*M ZnCl_2_ (Sigma-Aldrich) plus 4 *μ*M pyrithione (Sigma-Aldrich) or 10 *μ*M of the Zn^2+^-selective chelator N,N,N′,N′-tetrakis (2-pyridylmethyl) ethylenediamine (TPEN) (Sigma-Aldrich), respectively, added 10 min before OGD/R injury.

### 2.8. Detection of Mitochondrial Membrane Potential (ΔΨm) after H_2_O_2_ Injury

ΔΨm was assessed using confocal microscopy as described in previous studies [13, 16]. Briefly, H9c2 cardiac cells that had been pretreated with 1 *μ*M ZnCl_2_ plus 4 *μ*M pyrithione or 10 *μ*M TPEN for 10 mins were cultured in a specific temperature-controlled culture dish, and the cells were then exposed to 100 *μ*M H_2_O_2_ for 20 min to cause mitochondrial oxidant damage. Control cells were not pretreated but instead were incubated in DMEM without FBS. After these treatments, the cells were incubated with 100 nM tetramethylrhodamine ethyl ester (TMRE) (MolecularProbe) in standard Tyrode solution for 15 min and then mounted on the stage of a confocal microscope (Leica, Germany). The temperature was maintained at 37°C with a Delta T Open Dish System (Bioptechs, Butler, PA). The fluorescence intensity was determined based on 5 to 10 cells per computer-recorded image and quantified with Image Pro-Plus 5.1.

### 2.9. Upregulation of UQCRC1 by Diazoxide

To measure the mitochondrial free Zn^2+^ concentration in cells that overexpressed UQCRC1 or cells in which UQCRC1 was downregulated, H9c2 cells were stained with RhodZin-3, a Zn^2+^-selective fluorescence dye that targets mitochondria. The mitochondria-specific dye Mito-Tracker Green, which relies on green fluorescence as the specific fluorescence dye, was coloaded with the Zn^2+^-selective fluorescence dye RhodZin-3. Because this green fluorescence was very difficult to be distinguished from the green fluorescence of the Ad-UQCRC1 recombinant adenovirus, Ad-UQCRC1 cells could not be used for confocal imaging. Our previous study showed that delayed preconditioning by diazoxide upregulated UQCRC1 in rat myocardial cells (data not published). Therefore, we used long-term diazoxide treatment as an alternative method to up-regulate UQCRC1 expression. H9C2 cardiac cells were seeded in 96-well plates at a density of 3 × 10^3^ cells per well and subjected to different treatments: the control group was treated with solvent, and the DZ group was treated with 100 *μ*mol/L diazoxide for 48 hours. Subsequently, SDS-PAGE and Western blotting were performed to measure the level of UQCRC1 expression.

### 2.10. Downregulation of UQCRC1

Three siRNA duplexes targeting the rat UQCRC1 gene were designed using the targeted siRNA finder and design tool available at http://www.genscript.com/ and commercially obtained from Ribobio (Guangzhou, China). The siUQCRC1 silencer sequence (CCGUUGCUGUAGCUAACAAdTdT, 200 nmol/L) was identified and transfected into H9C2 cardiac cells. Western blotting was performed to quantify the expression of UQCRC1 and ensure that UQCRC1 expression was significantly downregulated in H9C2 cardiac cells.

### 2.11. Confocal Imaging of Mitochondrial Zn^2+^

The free zinc (Zn^2+^) concentration in the mitochondria of H9c2 cells was assessed with RhodZin-3 according to the manufacturer's instructions (MolecularProbe) and previous studies [29, 30]. After delayed preconditioning induced by diazoxide or 48 hours after siRNA transfection, H9c2 cells were cultured in a specific temperature-controlled culture dish. The dish was coloaded with both 5 *μ*M RhodZin-3 plus 0.02% pluronic acid (MolecularProbe) and 100 nM Mito-Tracker Green (Beyotime, China) in fresh medium without FBS for 30 min at 37°C in the dark in a humidified 5% CO_2_ + 95% air atmosphere. After 3 washes with phosphate-buffered saline, the cells were incubated for another 30 min for deesterification and then mounted on the stage of confocal microscope in a humidified 5% CO_2_ + 95% air atmosphere (Leica, Germany). The temperature was maintained at 37°C with a Delta T Open Dish System (Bioptechs, Butler, PA), and the fluorescence intensity was measured as described above with Image Pro-Plus 5.1.

### 2.12. Statistical Analysis

Continuous data are presented as the means ± SEM. The means of two groups were compared using independent Student's *t*-tests. Multiple groups were analyzed using an ANOVA with a Bonferroni post hoc test. Two-sided *P* values < 0.05 were considered to indicate a significant difference.

## 3. Results

### 3.1. UQCRC1 Overexpression Protects H9c2 Cardiac Cells from OGD/R and H2O2 Injury

**Figure 1:**
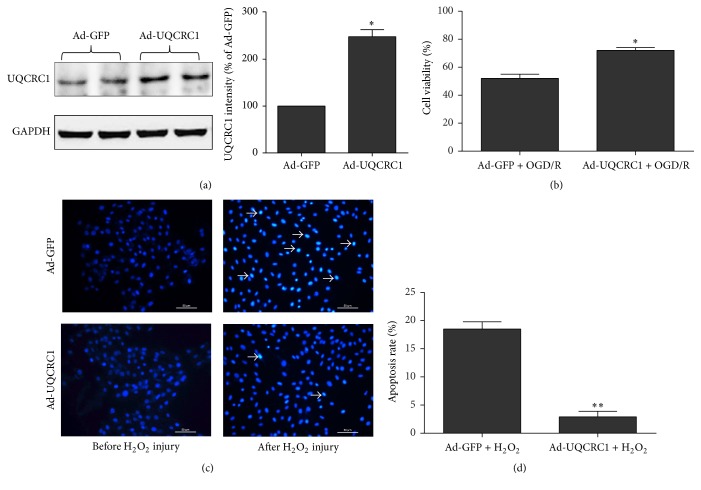
*UQCRC1 overexpression protected H9c2 cardiac cells from OGD/R and *H_2_O_2_* injury*. (a) UQCRC1 expression in H9c2 cardiac cells after transfection with Ad-GFP or Ad-UQCRC1 (*n* = 5). (b) The effect of UQCRC1 overexpression on the viability of H9C2 cardiac cells after OGD/R injury (*n* = 10). ((c) and (d)) The effect of UQCRC1 overexpression on the apoptosis rate of H9C2 cardiac cells after H_2_O_2_ injury (*n* = 10). Data are expressed as the means ± SEM; ^**∗**^*P* < 0.05 versus first group and ^**∗∗**^*P* < 0.01 versus the first group.

As shown in Figure 1(a), the Western blotting results showed that UQCRC1 expression was approximately 2.4 times higher in H9c2 cardiac cells transfected with Ad-UQCRC1 than in empty vector-transfected cells in the control group 48 h after transfection. This indicated that UQCRC1 protein in H9c2 cardiac cells could successfully be overexpressed by Ad-UQCRC1.

Subsequently, OGD/R and H_2_O_2_ injury models were established to examine the ability of UQCRC1 overexpression to protect H9c2 cardiac cells 48 h after transfection. Specifically, the cell viability was significantly higher in the UQCRC1 overexpression group (73% ± 2%) than in the control group (52% ± 3%, Figure 1(b)) after OGD/R injury, indicating that UQCRC1 overexpression could protect the cells. Moreover, necrotic cells were not evident in either the UQCRC1 overexpression or the control group before and after H_2_O_2_ injury. However, apoptotic H9c2 cells were observed in the two groups after H_2_O_2_ injury (Figure 1(c)). Furthermore, the results showed that, compared with the control group, UQCRC1 overexpression significantly attenuated apoptosis after H_2_O_2_ injury. Specifically, the apoptosis rate in UQCRC1 overexpression group was 2.9 ± 1.0%, which was significantly lower than that in control group (18.5 ± 1.3%). (Figure 1(d)).

### 3.2. Zinc Is an Indispensable Cofactor for the Protective Role of UQCRC1 Overexpression

**Figure 2:**
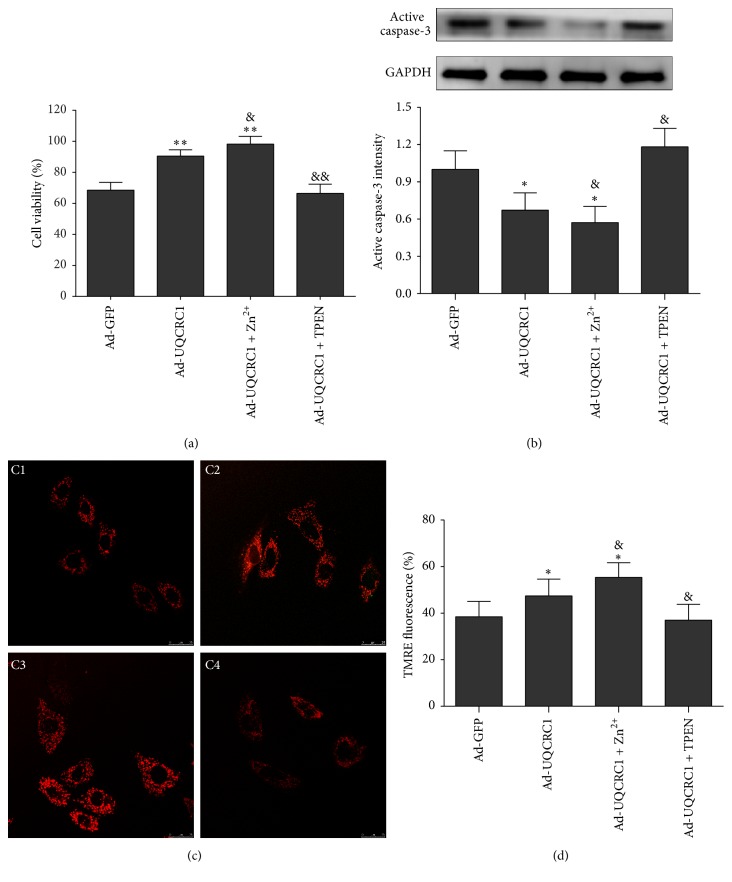
The effect of the interaction between UQCRC1 overexpression and Zn^2+^ on cardiac cell protection. (a) Viability of H9c2 cardiac cells after OGD/R injury (^**∗∗**^*P* < 0.01 versus Ad-GFP, ^&^*P* < 0.05 versus Ad-UQCRC1, and ^&&^*P* < 0.01 versus Ad-UQCRC1; *n* = 10). (b) Active caspase-3 expression in H9c2 cardiac cells assessed by Western blotting after OGD/R injury (^**∗**^*P* < 0.05 versus Ad-GFP and ^&^*P* < 0.05 versus Ad-UQCRC1; *n* = 3). (c) Confocal fluorescence images of TMRE (C1: Ad-GFP, C2: Ad-UQCRC1, C3: Ad-UQCRC1+Zn^2+^, and C4: Ad-UQCRC1+TPEN). (d) Mitochondrial membrane potential of H9c2 cells assessed by TMRE after H_2_O_2_ injury (summarized data for TMRE fluorescence intensity; ^*∗*^*P* < 0.05 versus Ad-GFP and ^&^*P* < 0.05 versus Ad-UQCRC1; *n* = 4). Data are expressed as the means ± SEM.

As shown in Figure 2(a), UQCRC1 overexpression significantly increased cell viability after OGD/R injury compared with the control group. Moreover, increasing the zinc concentration by directly adding Zn^2+^ significantly increased the cell viability in the Ad-UQCRC1+Zn^2+^ group compared to the Ad-UQCRC1 group. However, the addition of TPEN significantly decreased the cell viability in the Ad-UQCRC1+TPEN group compared to the Ad-UQCRC1 group and rendered the viability indistinguishable from that of the control group. This finding indicates that the increase in cell viability induced by UQCRC1 overexpression was supported by Zn^2+^ but abrogated by Zn^2+^-selective chelator TPEN.

Additionally, the expression of active caspase-3 in H9c2 cardiac cells after OGD/R injury was decreased by UQCRC1 overexpression (Figure 2(b)), and this decrease was further strengthened by the addition of Zn^2+^. Conversely, the addition of TPEN abrogated this decrease in active caspase-3. The mitochondrial membrane potential (ΔΨm) of H9c2 cardiac cells was then assessed with tetramethylrhodamine ethyl ester (TMRE) (Figure 2(c)). As shown in Figure 2(d), H_2_O_2_ injury resulted in a loss of ΔΨm, which was attenuated by UQCRC1 overexpression. Furthermore, exogenous Zn^2+^ markedly enhanced the effect of UQCRC1 overexpression-mediated maintenance of the ΔΨm, whereas TPEN completely reversed this effect. These findings suggest that UQCRC1 overexpression contributes to maintaining the mitochondrial function of H9c2 cardiac cells during injury and that Zn^2+^ plays an important and indispensable role in this effect.

### 3.3. UQCRC1 Expression Changes Directly Influence the Mitochondrial Free Zn^2+^ Concentration

**Figure 3:**
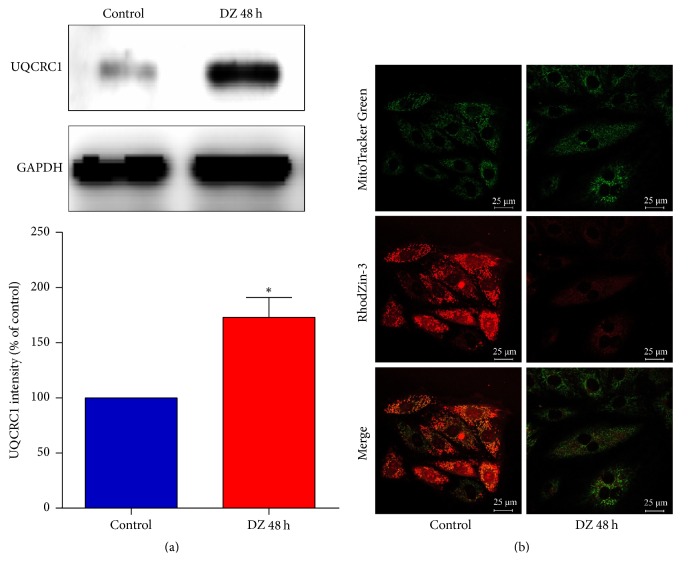
The effect of UQCRC1 upregulation on the mitochondrial free Zn^2+^ concentration. (a) The expression of UQCRC1 in H9c2 cells after diazoxide treatment measured by Western blotting (data are expressed as the means ± SEM; ^*∗*^*P* < 0.05 versus control, *n* = 4). (b) Confocal fluorescence images various groups of H9c2 cells loaded with both Mito-Tracker Green and RhodZin™-3.

**Figure 4:**
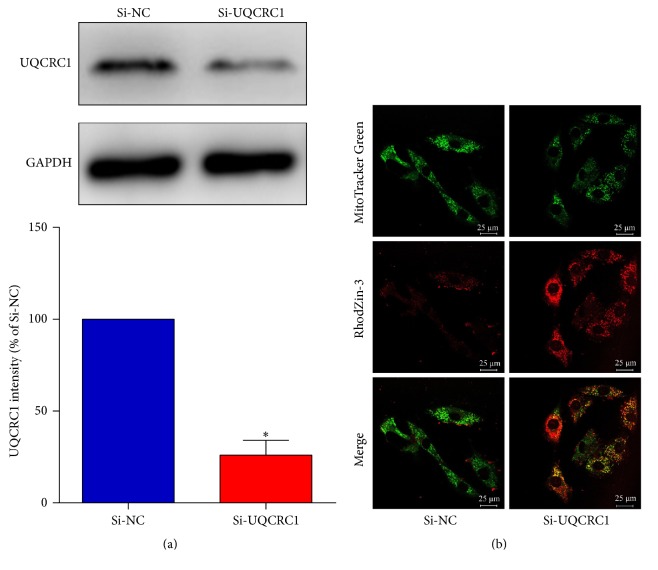
The effect of UQCRC1 downregulation on the mitochondrial free Zn^2+^ concentration. (a) The expression of UQCRC1 in H9c2 cells as detected by Western blotting after transfection (data are expressed as the means ± SEM; ^*∗*^*P* < 0.05 versus control, *n* = 4). (b) Confocal fluorescence images of H9c2 cells in various groups loaded with both Mito-Tracker Green and RhodZin-3.

As shown in Figure 3(a), 48 hours of diazoxide treatment upregulated UQCRC1 in H9c2 cardiac cells. We also measured Zn^2+^ concentration and found that the mitochondrial free Zn^2+^ fluorescence intensity was significantly decreased in the UQCRC1 upregulation group compared with the control group (Figure 3(b)). This finding indicated that the upregulation of UQCRC1 decreased the mitochondrial free Zn^2+^ concentration. As shown in Figure 4(a), UQCRC1 protein expression was significantly downregulated after transfection with UQCRC1 siRNA (Figure 4(a)). As shown in Figure 4(b), the mitochondrial free Zn^2+^ fluorescence intensity was significantly increased in si-UQCRC1 group compared with control siRNA group, indicating that the downregulation of UQCRC1 increased the mitochondrial free Zn^2+^ concentration.

## 4. Discussion

In this study, we explored the role of UQCRC1 overexpression in protecting H9c2 cardiac cells against OGD/R and peroxidation injury. We also investigated the possible mechanism underlying the UQCRC1 overexpression-mediated cardiac protection by focusing on mitochondrial Zn^2+^. The results of this study provide direct evidence for the cardio-protective role of UQCRC1 overexpression, which is likely mediated by mitochondrial Zn^2+^.

The H9c2 cardiac cells are commonly used to mimic myocardial damage and have been used in many previous studies, especially in those studies that explore the integration between some targeted proteins and Zn^2+^ [13, 16, 24, 28]. Therefore, H_2_O_2_ and OGD/R induced injury models in H9c2 cells were chosen in the study. In the current study, we observed that several injury markers, including the apoptosis rate, cell viability, and expression of active caspase-3, were significantly increased after the H_2_O_2_ and OGD/R injury. However, the upregulation of UQCRC1 with a recombinant adenovirus vector containing UQCRC1 significantly reduced injury to H9c2 cells.

UQCRC1 expression changes have been detected in several previous studies. For example, Lin et al. used a proteomic approach and found that the expression levels of six proteins involved in energy metabolism, including UQCRC1, decreased after I/R injury in isolated rat hearts [5]. In another proteomic study [2], UQCRC1 was found to increase in response to the cardio-protective effect of pharmacological preconditioning with GSK inhibitor VIII. Furthermore, the current results showed that the apoptosis rate and the expression of active caspase-3 were decreased and the cell viability was increased in H9c2 cells that overexpressed UQCRC1. Therefore, these results indicate that UQCRC1 overexpression may protect H9c2 cells from OGD/R injury.

UQCRC1 gene overexpression is known to enhance complex III activity in mice [9]. Furthermore, mitochondrial dysfunction was recently associated with a specific loss of UQCRC1 in cells of epithelial origin [10] and mouse spermatocytes [31]. These studies suggest that UQCRC1 plays an important role in the mitochondrial function. Interestingly we also found that UQCRC1 overexpression significantly attenuated the loss of ΔΨm, an indicator of mitochondrial function, after H_2_O_2_ injury. Therefore, UQCRC1 overexpression contributes to maintaining mitochondrial function in H9c2 cells during injury and may protect cardiac cells.

Recently, several studies have shown that the function of UQCRC1 might be far more extensive than previously implied, particularly because of its notable role in oncology. UQCRC1 was demonstrated to be highly expressed in breast and ovarian tumors [32] as well as in osteosarcoma cells and tissues [33]. In addition, some anticancer treatments, for example, treatment of CAL-27 cells with 11-dehydrosinulariolide and A375 melanoma cells with sinulariolide, markedly downregulate UQCRC1 [34, 35]. These studies strongly suggest that UQCRC1 overexpression may promote proliferation and inhibit apoptosis in cancer cells, which also suggests that UQCRC1 can protect cells from injury. From several viewpoints, the current study showed that UQCRC1 overexpression protected H9c2 cardiac cells from OGD/R and H_2_O_2_ injury. These results also indicate that a normal expression of UQCRC1 is necessary for the cardioprotection. Based on our data and the aforementioned previous studies, we conclude that UQCRC1 plays an important role in cardiac protection.

In many previous studies, Zn^2+^ has been reported to play a cardio-protective role in IPC, postconditioning, or pharmacological preconditioning [13–17]. Although the specific mechanism by which Zn^2+^ protects cardiac cells remains controversial [13, 14, 16], the antiapoptotic effect of Zn^2+^ was attributed to the maintenance of mitochondrial function and a direct suppression of caspase-3 [13, 14, 36, 37], which is consistent with the results of the current study. In the current study, we first used exogenous Zn^2+^ and the membrane-permeable Zn^2+^-selective chelator TPEN to investigate the possible relationship between Zn^2+^ and the cardio-protective role of UQCRC1 overexpression. The results showed that exogenous Zn^2+^ markedly enhanced the protective effects of UQCRC1 overexpression, as indicated by increased cell viability, suppressed caspase-3 expression, and the maintenance of ΔΨm after injury, and these protective effects were completely abolished when TPEN was applied. These results demonstrated that Zn^2+^ significantly affected the protective effects of UQCRC1 overexpression in H9c2 cardiac cells.

Although the above findings strongly suggest that Zn^2+^ is a required cofactor for the protective role of UQCRC1 overexpression, whether this protective effect was due to their combined function remained unclear. Several early studies demonstrated that zinc binds to residues in the vicinity of the iron-sulfur protein (ISP) [19, 20], and UQCRC1 is located near the NH_2_- terminus of the ISP [12]. Thus, UQCRC1 may contain Zn^2+^ binding site. To explore the association between UQCRC1 expression and Zn^2+^ concentration in mitochondria, we stained H9c2 cells with RhodZin-3, a Zn^2+^-selective fluorescence dye that targets the mitochondria, to measure the mitochondrial free Zn^2+^ concentration in cells that overexpress UQCRC1 and cells in which UQCRC1 was downregulated. The results showed that the upregulation of UQCRC1 reduced the concentration of free Zn^2+^ in mitochondria, whereas downregulation of UQCRC1 increased concentration of free Zn^2+^, indicating that UQCRC1 may bind Zn^2+^ in mitochondria. Therefore, changes in UQCRC1 expression directly influence the mitochondrial free Zn^2+^ concentration. Based on these results, we speculated that UQCRC1 overexpression protects H9c2 cardiac cells from ischemia/reperfusion injury by binding zinc.

Several limitations should be considered for the current study. First, the protective effect of UQCRC1 overexpression was only evaluated at the cellular level in H9c2 cells, which are commonly used but under debate as an in vitro cell model, and thus further in vivo models are needed to validate the current results. Second, only overexpression and downregulation models were applied in the current study to explore the role and mechanism of UQCRC1 in cardio-protection, and UQCRC1 knockout model that completely abrogates UQCRC1 expression should be considered in future studies to confirm this effect.

## 5. Conclusions

In summary, the current study showed that the overexpression of UQCRC1 can protect H9c2 cardiac cells against OGD/R injury, which provides direct evidence for the cardio-protective role of UQCRC1. These cardio-protective effects were significantly enhanced by exogenous Zn^2+^ but completely abolished by the Zn^2+^-selective chelator TPEN. Furthermore, the upregulation of UQCRC1 decreased the concentration of free Zn^2+^ in mitochondria, whereas the downregulation of UQCRC1 increased this concentration. Thus, UQCRC1 may play an important role in cardiac protection by binding with zinc.

## References

[1] Yellon D. M., Hausenloy D. J. (2007). Myocardial reperfusion injury. *The New England Journal of Medicine*.

[2] Wong R., Aponte A. M., Steenbergen C., Murphy E. (2010). Cardioprotection leads to novel changes in the mitochondrial proteome. *American Journal of Physiology - Heart and Circulatory Physiology*.

[3] Cabrera J. A., Butterick T. A., Long E. K. (2013). Reduced expression of mitochondrial electron transport chain proteins from hibernating hearts relative to ischemic preconditioned hearts in the second window of protection. *Journal of Molecular and Cellular Cardiology*.

[4] McLeod C. J., Jeyabalan A. P., Minners J. O., Clevenger R., Hoyt R. F., Sack M. N. (2004). Delayed ischemic preconditioning activates nuclear-encoded electron-transfer-chain gene expression in parallel with enhanced postanoxic mitochondrial respiratory recovery. *Circulation*.

[5] Lin H.-B., Cadete V. J. J., Sawicka J., Wozniak M., Sawicki G. (2012). Effect of the myosin light chain kinase inhibitor ML-7 on the proteome of hearts subjected to ischemia-reperfusion injury. *Journal of Proteomics*.

[6] Chen Q., Vazquez E. J., Moghaddas S., Hoppel C. L., Lesnefsky E. J. (2003). Production of reactive oxygen species by mitochondria: central role of complex III. *Journal of Biological Chemistry*.

[7] Christians E. S., Benjamin I. J. (2012). Proteostasis and REDOX state in the heart. *American Journal of Physiology—Heart and Circulatory Physiology*.

[8] Zhang L., Jaswal J. S., Ussher J. R. (2013). Cardiac insulin-resistance and decreased mitochondrial energy production precede the development of systolic heart failure after pressure-overload hypertrophy. *Circulation: Heart Failure*.

[9] Kriaucionis S., Paterson A., Curtis J., Guy J., MacLeod N., Bird A. (2006). Gene expression analysis exposes mitochondrial abnormalities in a mouse model of Rett syndrome. *Molecular and Cellular Biology*.

[10] Shibanuma M., Inoue A., Ushida K. (2011). Importance of mitochondrial dysfunction in oxidative stress response: A comparative study of gene expression profiles. *Free Radical Research*.

[11] Perrelli M. G., Pagliaro P., Penna C. (2011). Ischemia/reperfusion injury and cardioprotective mechanisms: role of mitochondria and reactive oxygen species. *World Journal of Cardiology*.

[12] Iwata S., Lee J. W., Okada K. (1998). Complete structure of the 11-subunit bovine mitochondrial cytochrome bc1 complex. *Science*.

[13] Chanoit G., Lee S., Xi J. (2008). Exogenous zinc protects cardiac cells from reperfusion injury by targeting mitochondrial permeability transition pore through inactivation of glycogen synthase kinase-3*β*. *American Journal of Physiology—Heart and Circulatory Physiology*.

[14] Jang Y., Wang H., Xi J., Mueller R. A., Norfleet E. A., Xu Z. (2007). NO mobilizes intracellular Zn2+ via cGMP/PKG signaling pathway and prevents mitochondrial oxidant damage in cardiomyocytes. *Cardiovascular Research*.

[15] McIntosh R., Lee S., Ghio A. J. (2010). The critical role of intracellular zinc in adenosine A2 receptor activation induced cardioprotection against reperfusion injury. *Journal of Molecular and Cellular Cardiology*.

[16] Lee S., Chanoit G., McIntosh R., Zvara D. A., Xu Z. (2009). Molecular mechanism underlying Akt activation in zinc-induced cardioprotection. *American Journal of Physiology - Heart and Circulatory Physiology*.

[17] Xi J., Tian W., Zhang L., Jin Y., Xu Z. (2010). Morphine prevents the mitochondrial permeability transition pore opening through NO/cGMP/PKG/Zn2+/GSK-3*β* signal pathway in cardiomyocytes. *American Journal of Physiology - Heart and Circulatory Physiology*.

[18] Beharier O., Dror S., Levy S. (2012). ZnT-1 protects HL-1 cells from simulated ischemia-reperfusion through activation of Ras-ERK signaling. *Journal of Molecular Medicine*.

[19] Lorusso M., Cocco T., Sardanelli A. M., Minuto M., Bonomi F., Papa S. (1991). Interaction of Zn2+ with the bovine‐heart mitochondrial bc1 complex. *European Journal of Biochemistry*.

[20] Link T. A., Von Jagow G. (1995). Zinc ions inhibit the QP center of bovine heart mitochondrial bc1 complex by blocking a protonatable group. *Journal of Biological Chemistry*.

[21] Merten K. E., Jiang Y., Feng W., Kang Y. J. (2006). Calcineurin activation is not necessary for doxorubicin-induced hypertrophy in H9c2 embryonic rat cardiac cells: involvement of the phosphoinositide 3-kinase-akt pathway. *Journal of Pharmacology and Experimental Therapeutics*.

[22] Zhao W., Waggoner J. R., Zhang Z.-G. (2009). The anti-apoptotic protein HAX-1 is a regulator of cardiac function. *Proceedings of the National Academy of Sciences of the United States of America*.

[23] Lu N., Sun Y., Zheng X. (2011). Orientin-induced cardioprotection against reperfusion is associated with attenuation of mitochondrial permeability transition. *Planta Medica*.

[24] Ho J. C., Wu S., Kam K. W. L., Sham J. S. K., Wong T. M. (2002). Effects of pharmacological preconditioning with U50488H on calcium homeostasis in rat ventricular myocytes subjected to metabolic inhibition and anoxia. *British Journal of Pharmacology*.

[25] Shin E.-J., Schram K., Zheng X.-L., Sweeney G. (2009). Leptin attenuates hypoxia/reoxygenation-induced activation of the intrinsic pathway of apoptosis in rat H9c2 cells. *Journal of Cellular Physiology*.

[26] Hashimoto D., Ohmuraya M., Hirota M. (2008). Involvement of autophagy in trypsinogen activation within the pancreatic acinar cells. *Journal of Cell Biology*.

[27] Chen Y. L., Zhuang X. D., Xu Z. W. (2013). Higenamine combined with [6]-gingerol suppresses doxorubicin-triggered oxidative stress and apoptosis in cardiomyocytes via upregulation of PI3K/Akt pathway. *Evidence-based Complementary and Alternative Medicine*.

[28] Song J., Guo X., Xie X. (2011). Autophagy in hypoxia protects cancer cells against apoptosis induced by nutrient deprivation through a beclin1-dependent way in hepatocellular carcinoma. *Journal of Cellular Biochemistry*.

[29] Rudolf E., Rudolf K., Cervinka M. (2005). Zinc induced apoptosis in HEP-2 cancer cells: The role of oxidative stress and mitochondria. *BioFactors*.

[30] Sensi S. L., Ton-That D., Weiss J. H., Rothe A., Gee K. R. (2003). A new mitochondrial fluorescent zinc sensor. *Cell Calcium*.

[31] Huang S., Wang J., Cui Y. (2016). 2,2′,4,4′-Tetrabromodiphenyl ether injures cell viability and mitochondrial function of mouse spermatocytes by decreasing mitochondrial proteins Atp5b and Uqcrc1. *Environmental Toxicology and Pharmacology*.

[32] Kulawiec M., Arnouk H., Desouki M. M., Kazim L., Still I., Singh K. K. (2006). Proteomic analysis of mitochondria-to-nucleus retrograde response in human cancer. *Cancer Biology and Therapy*.

[33] Liu X., Zeng B., Ma J., Wan C. (2009). Comparative proteomic analysis of osteosarcoma cell and human primary cultured osteoblastic cell. *Cancer Investigation*.

[34] Liu C.-I., Chen C.-C., Chen J.-C. (2011). Proteomic analysis of anti-tumor effects of 11-dehydrosinulariolide on CAL-27 cells. *Marine Drugs*.

[35] Li H.-H., Su J.-H., Chiu C.-C. (2013). Proteomic investigation of the sinulariolide-treated melanoma cells A375: Effects on the cell apoptosis through mitochondrial-related pathway and activation of caspase cascade. *Marine Drugs*.

[36] Truong-Tran A. Q., Carter J., Ruffin R. E., Zalewski P. D. (2001). The role of zinc in caspase activation and apoptotic cell death. *BioMetals*.

[37] Perry D. K., Smyth M. J., Stennicke H. R. (1997). Zinc is a potent inhibitor of the apoptotic protease, caspase-3: a novel target for zinc in the inhibition of apoptosis. *Journal of Biological Chemistry*.

